# Effects of hydrogen on microstructure evolution and mechanical properties of TB8 titanium alloy

**DOI:** 10.1371/journal.pone.0297528

**Published:** 2025-01-16

**Authors:** Yequan Chen, Shiying Wang, Jiaxing Meng, Naotoshi Mitsuzaki, Zhidong Chen

**Affiliations:** 1 School of Materials Science and Engineering, Changzhou University, Changzhou, China; 2 Qualtec Co, Ltd, Osaka, Japan; 3 School of Petrochemical Engineering, Changzhou University, Changzhou, China; Semnan University, ISLAMIC REPUBLIC OF IRAN

## Abstract

The influence of varying hydrogen content on the microstructure, mechanical properties, and fracture behavior of the metastable β titanium alloy TB8 after hydrogen charging has been investigated in this study. Several characterization methods, including optical microscopy (OM), x-ray diffraction (XRD), scanning electron microscopy (SEM), and transmission electron microscopy (TEM), were employed to comprehensively analyze the alloy. The results show that with the addition of hydrogen, hydrogen mainly accumulated at grain boundaries in the form of hydrides. The β phase diffraction peak shifted to a lower angle, which can be attribute to hydrogen-induced lattice distortion. As the hydrogen content increases, γ-TiH hydrides and ε-TiH_2_ hydrides were observed. Ultimate tensile strength of the alloy firstly increased from 982 MPa to 1636 MPa, and then decreased to 1432 MPa. Uniform elongation decreased from 33% to 19%. Fracture mode transitioned from ductile to brittle with increasing hydrogen. In summary, we hope the outcome of this work could provide important insights toward the hydrogen charging influences the microstructure, mechanical properties, and fracture behavior of the TB8 titanium alloy.

## 1. Introduction

Titanium alloys can be classified into alpha (α) and near alpha (α/β) alloys, as well as beta (β) alloys, based on their phases. β alloys are further categorized into stable and metastable alloys. Stable alloys exhibit a full β phase microstructure at room temperature, while metastable alloys experience α phase precipitation within the β matrix during air cooling or quenching aging [1].

Titanium and its alloys are widely used in important sectors such as automobile manufacturing, military equipment, marine engineering, chemical industry, and aerospace due to their excellent corrosion resistance, high specific strength, and good fatigue performance [2–5]. Titanium is highly corrosion-resistant and possesses excellent hydrogen storage properties [6, 7]. However, its strong affinity for hydrogen makes it susceptible to hydrogen absorption. Hydrogen has a very low solubility in α titanium, with a solubility of 0.15 wt% in the α Ti phase at 300°C. Even a small amount of hydrogen can render titanium brittle, thus significantly impairing its suitability for structural applications [8–10]. Hydrogen embrittlement in titanium is primarily caused by the dissolution and diffusion of hydrogen within the titanium matrix, leading to the formation of hydrides [11–14], which can be attributed to the interaction between hydrogen and defects [15]. Hydrogen preferentially concentrates in regions of local stress concentration, such as vacancies, dislocations, precipitates, phases, and grain boundaries, resulting in lattice expansion and distortion [16]. Previous studies have demonstrated spontaneous hydrogen absorption reactions between titanium alloys, leading to the formation of hydrides and the dissolved of hydrogen in the α and β phases of titanium alloys. When the hydrides content exceeds a critical level, enhanced hydrogen embrittlement occurs, resulting in a considerable decrease in ductility and a transition from dimple fracture to cleavage fracture.

The Previous studies have identified three types of hydrides in titanium alloys: δ, ε, and γ hydrides. The γ hydride with an ordered arrangement of hydrogen atoms has a body-centered tetragonal (bct) structure, which is formed at low hydrogen concentrations. As the dissolved hydrogen content increases, the δ hydride exhibits an fcc unit cell with hydrogen atoms occupying the tetrahedral interstitial sites, forming a CaF_2_-type structure. When the concentration of dissolved hydrogen atoms exceeds a certain threshold, ε-TiH_2_ hydride, characterized by a bct (c/a < 1) structure, precipitates from the α phase matrix. Titanium alloys with high sensitivity to hydrogen embrittlement are susceptible to loss of ductility and hydrogen-induced delayed cracking. Consequently, extensive study has aimed to elucidate the fracture mechanism of Ti-based materials in hydrogen-rich environments. According to Ref [17], the diffusivity and solubility of hydrogen in the α and β phases of titanium alloys differ, usually associated with the number of interstitial sites, such as tetrahedral and octahedral sites, within their duplex structures [18–20]. These studies suggest that hydrogen prefers diffusion within the β lattice and subsequent reaction with the α phase along the α/β interface when the proportion of β phase is sufficiently high. Recent work has also indicated that the formation and cracking of δ and ε hydrides primarily occur within the α phase or along the α/β interface [21]. The α/β interface, as an effective trapping site for hydrogen, is considered the primary propagation pathway for brittle failure [22].

Previous study on TiHx mainly focused on the hydrogen embrittlement behavior of α-Ti and (α + β) dual-phase Ti alloys, with relatively fewer studies conducted on β type titanium alloys. Therefore, this study investigates the effect of hydrogen charging on the microstructure evolution and mechanical behavior of the metastable TB8 titanium alloy. TB8 alloy is a metastable β type titanium alloy characterized by high specific strength, excellent cold formability, improved oxidation and corrosion resistance, elevated temperature strength, and good thermal stability. It is applicable to structural components across several industries. Aerospace: aircraft frames and structural components, engine components, missile and spacecraft structural elements. Medical: Orthopedic implants, surgical instruments and dental implants. Automotive: engine parts, exhaust systems and suspension components. Marine: boat and ship components, offshore drilling equipment and marine engine parts. Chemical Processing: pressure vessels, heat exchangers, and reactor components. However, components used in such applications are inevitably exposed to hydrogen gas. Hence, understanding the forms and mechanisms of hydrogen in titanium constitutes a vital scientific issue for the long-term use of titanium-based materials within hydrogen environments [23]. It is of great significance to comprehend the influence of hydrogen on the properties of titanium alloys, anticipate and address concerns related to hydrogen embrittlement. The novelty of our work can be summarized as:

- Various analytical techniques including OM, XRD, SEM, and TEM are conducted in this research.
- The effect of hydrogen charging on the microstructure evolution and mechanical behavior of the metastable TB8 titanium alloy are studied in this paper.
- Elucidated the effect of hydrogen on the microstructure, mechanical properties and fracture behavior of metastable β-titanium alloy TB8, providing valuable insights into the development of hydrogen-resistant titanium alloys.

This study employed various characterization techniques, including OM, XRD, SEM, and TEM to investigate the influence of hydrogen charging on the microstructure evolution and mechanical properties of metastable TB8 titanium alloy.

## 2. Methodology

The metastable β type TB8 titanium alloy used in this study is obtained from Baoji Hengxin Derui Metal Material Co., Ltd., and its chemical composition is presented in Table 1. Initially, a section is excised from the TB8 titanium alloy ingot, and subsequent sheet formation is achieved through the rolling process. The resulting material is subjected to dual annealing procedures, commencing with a 2-hour treatment at 830°C, followed by a 6-hour annealing at 560°C. In this study, high-temperature gas charge is employed, which involves precise control of hydrogen pressure, gas flow rate, and charging temperature to achieve the desired hydrogen content. This method allows for even distribution of hydrogen and high charging efficiency. The hydrogen charging test is conducted below the β transformation temperature of TB8 titanium alloy, which is 815°C. Initially, the titanium plate is wire cut into thin plates measuring 84 mm × 36 mm × 3 mm. Mechanical polishing with 400, 800, 1200, 1500, 2000, and 3000 sandpaper was performed to remove the oxide and surface impurities. Subsequently, the samples are ultrasonically cleaned in an acetone solution and dried. The cleaned samples are then placed into a custom-made hydrogen charging tube evacuated of air and inserted into a tube furnace. Once the temperature of the hydrogen charging tube stabilized at the charging temperature of 500°C, a specific hydrogen flow at a constant pressure (99.999% purity) is introduced. After reaching the desired hydrogen content, the samples are cooled in the furnace. Finally, samples with hydrogen contents of 0.02 wt%, 0.06 wt%, 0.10 wt%, 0.14 wt%, and 0.18 wt% are obtained. In this experiment, the etchant used for metallographic observation of TB8 titanium alloy with varying hydrogen content was Kroll’s reagent (1ml HF, 3ml HNO3, and 96ml H2O), which was also employed in accordance with the etchant used in the Ref [24].

**Table 1:** Chemical composition of as-cast TB8 titanium alloy (wt%).

Element	Ti	Mo	Al	Si	Fe	Nb	N	H	O	Others
Amount	Balance	15.58	2.63	0.22	0.28	2.62	0.004	0.001	0.09	0.4

**Fig 1:**
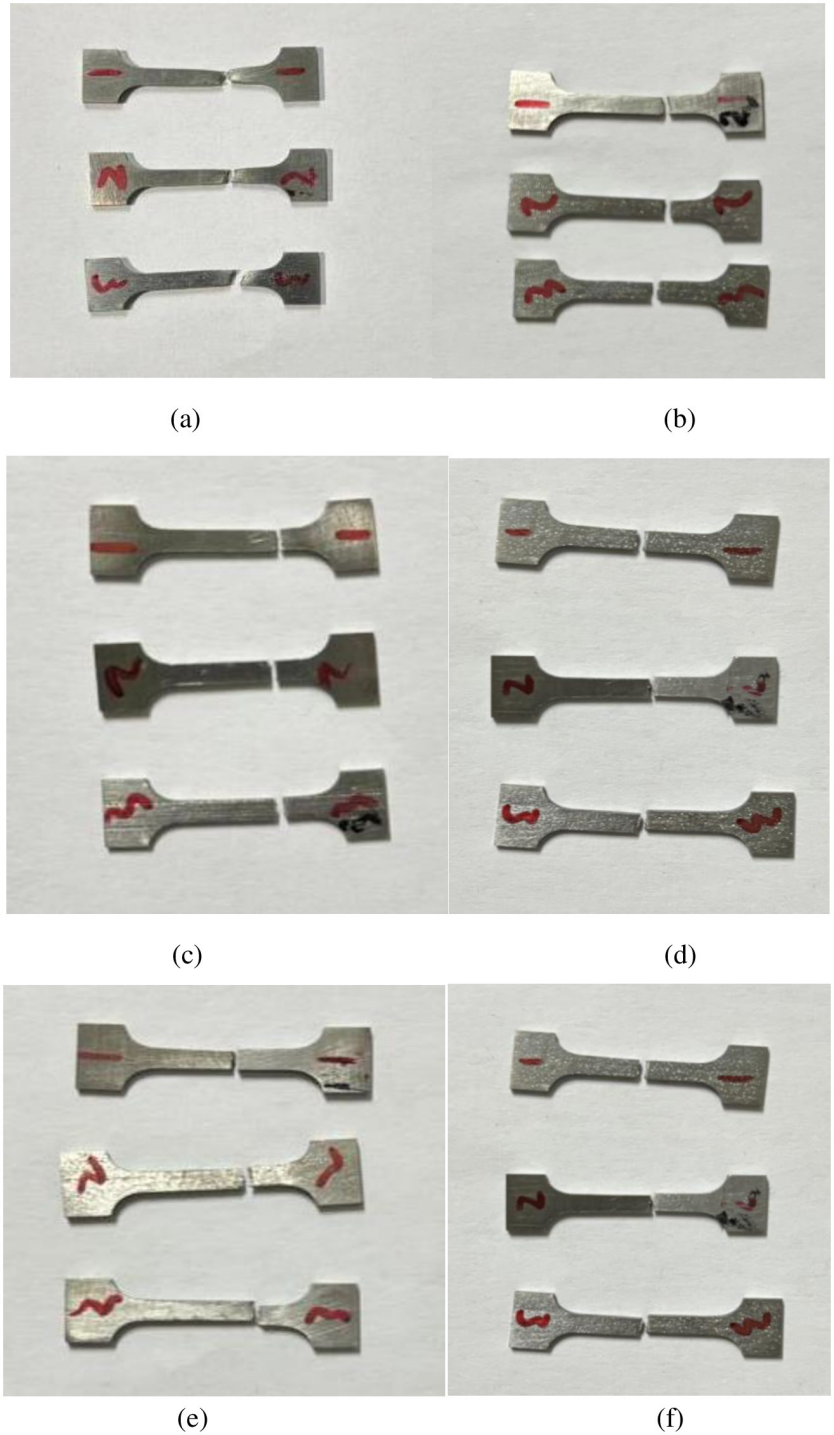
Tensile specimens of TB8 titanium alloy. (a) Without hydrogen content; (b) With hydrogen content of 200ppm; (c) With hydrogen content of 600ppm; (d) With hydrogen content of 1000ppm; (e) With hydrogen content of 1400ppm; (f) With hydrogen content of 1800ppm.

The phase identification of the samples is performed using Cu Kα radiation X-ray diffraction (XRD) with a D/MAX2500VL X-ray diffractometer. The XRD analysis is conducted on both the hydrogen charged samples and the original samples. The XRD testing parameters for this experiment were set based on the parameters outlined in the Ref [25]. In the literature, the scanning speed was specified as 3°/min. However, for increased precision in this current experiment, the scanning speed was adjusted to 1°/min. Additionally, the acceleration voltage was set at 40 kV, the current at 40 mA, and the scanning range from 20 to 90°. The metallographic microstructure of the titanium alloy before and after hydrogen charging is examined using a PG-2C optical microscope (OM). The samples are polished sequentially with 400, 800, 1000, 1200, 1500, 2000, and 3000 metallographic sandpaper. Diamond polishing paste was then used on the polishing machine, followed by etching the samples using Kroll’s reagent (1ml HF, 3ml HNO_3_, and 96ml H_2_O). It is observed that the hydrogen content significantly affected the corrosion effect, necessitating appropriate adjustments to the proportion of the corrosion solution to obtain clear metallographic structures. The metallographic structure is observed under a BH-200M inverted metallographic microscope. For the sample with a hydrogen content of 0.18 wt%, JEOL 2100 transmission electron microscopy (TEM) is employed. The preparation of transmission samples involved sanding the sample to a thickness of approximately 80 μm, followed by ion thinning for optimal imaging. The tensile experiment in this study referred to the tensile parameters mentioned in the Ref [26], where the tensile rate was 1×10−^4^/s. In this experiment, room temperature tensile testing was conducted, with a corresponding tensile rate of 1×10−^4^/s. Room temperature tensile tests are conducted using an LFM-20KG microcomputer-controlled electronic universal testing machine. To minimize testing errors, samples with different hydrogen contents underwent three rounds of stretching. The tensile fracture morphology is explored using a JSM-6360LA scanning electron microscope (SEM). Tensile specimens of TB8 titanium alloy without hydrogen filling and with different hydrogen contents are provided in Fig 1.

The microhardness was measured using an HXD-1000TMSC/LCD Vickers microhardness tester under a 0.98 N load for a duration of 10 seconds, set up according to Ref [27]. To reduce errors, ten measurements are taken at different positions for samples, and the maximum and minimum values are discarded. The average value is considered the final microhardness measurement.

## 3. Results and discussion

### 3.1 Effects of hydrogen on alloy microstructure

**Fig 2:**
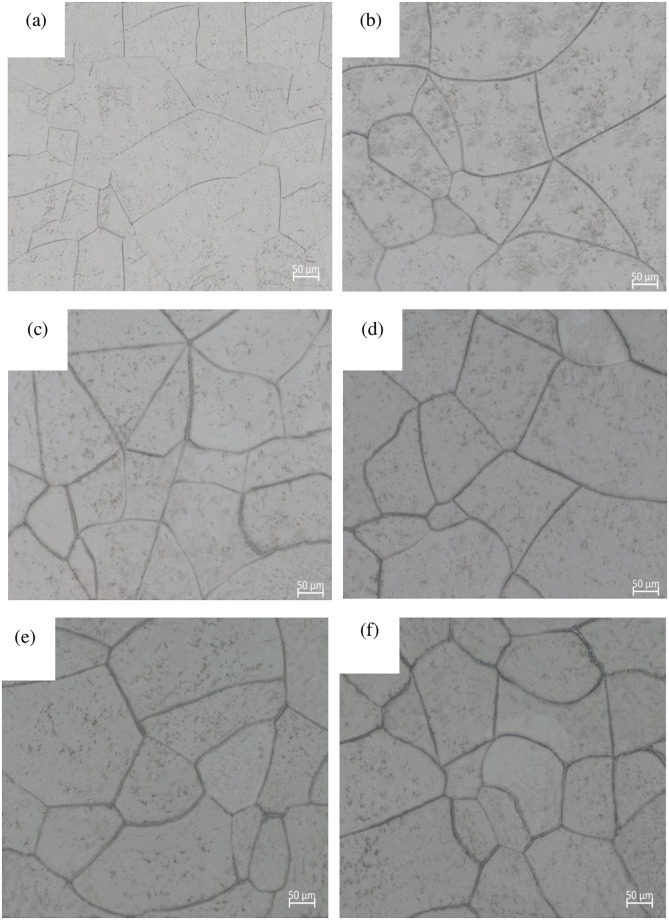
Microstructure of as-received and different hydrogen content of TB8 titanium alloy: (a) as-received; (b) 0.02 wt%H; (c) 0.06 wt%H; (d) 0.10 wt%H; (e) 0.14 wt%H; (f) 0.18 wt%H.

In order to compare with samples with different hydrogen charging amounts, the original samples were subjected to the same heat treatment as the hydrogen charged samles in Ar, resulting in an alloy with a hydrogen content of 0 wt%. Fig 2(a) displays the metallographic structure of the TB8 titanium alloy with 0 wt% hydrogen content. The original structure comprises β grains and fine α scattered in the β matrix. The β grains appear polygonal rather than equiaxed.

**Fig 3:**
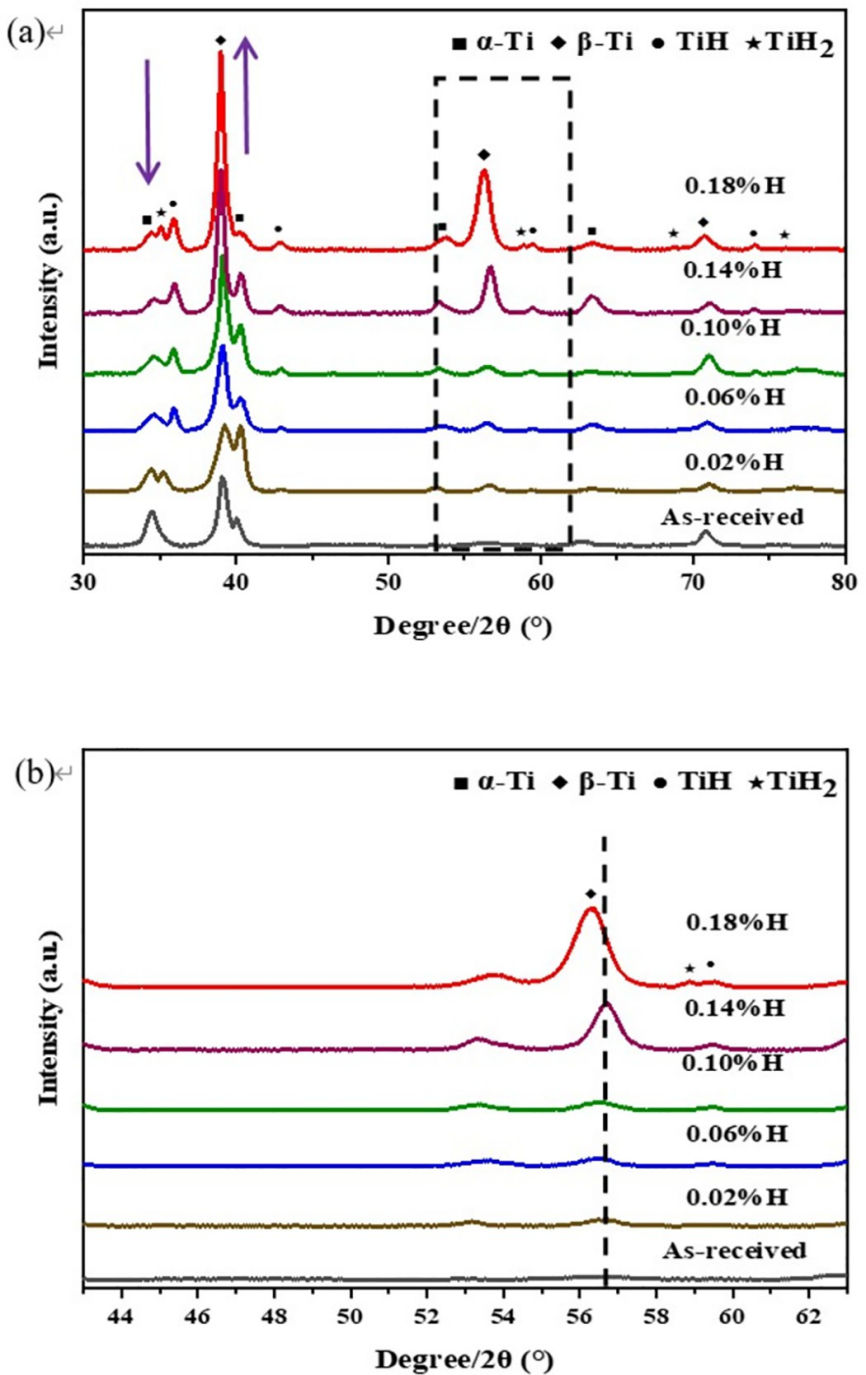
(a) XRD patterns of Ti alloy after charging for various conditions; (b) section of XRD patterns in (a) ranging from 53°to 63°.

When examining the metallographic structure with 0.02 wt% hydrogen content in Fig 2(b), significant changes in the structure after hydrogen charging can be observed compared to the original structure. Additional black precipitates develop within the β matrix and along grain boundaries. The XRD pattern in Fig 3 confirms the appearance of two different types of hydrides: the δ hydride with an fcc (face-centered cubic) structure and the ε hydride with a bct (body centered tetragonal) structure. The observed black material is a mixture of the α phase and hydrides, with hydrides precipitating at the grain boundaries. As the hydrogen content increases, the number of α phases on the β matrix gradually decreases due to the α to β phase transition by the presence of hydrogen [28].

The XRD spectra comparison between the original sample and the hydrogen charged samples is depicted in Fig 3. Fig 3(a) displays the XRD spectrum of the hydrogen charged and uncharged samples, and Fig 2b shows the section of XRD analyses in Fig 3(a) as marked by the black dashed box. The sample with 0 wt% hydrogen content predominantly consists of the α phase with a hexagonal close-packed (hcp) structure and the β phase with a body-centered cubic (bcc) structure. The XRD pattern of the hydrogen charged sample exhibits significant changes. As the hydrogen content increases, the relative intensity of the β phase diffraction peak gradually rises, while the relative intensity of the α phase diffraction peak decreases, as indicated by the arrow in the figure. This trend is consistent with previous literature reports [29]. Hydrogen acts as a β phase stabilizing element, enhancing the β phase stability in the TB8 titanium alloy at high temperatures and reducing the β transition temperature and critical transformation rate of the alloy [30, 31]. The introduction of hydrogen induces an α→β transition during the hydrogen charging process, resulting in an increased proportion of the β phase and a decreased proportion of the α phase. At the same time, the addition of hydrogen causes the β-phase lattice to expand, causing the β peak to shift to a lower angle [28], as shown in Fig 3(b).

**Fig 4:**
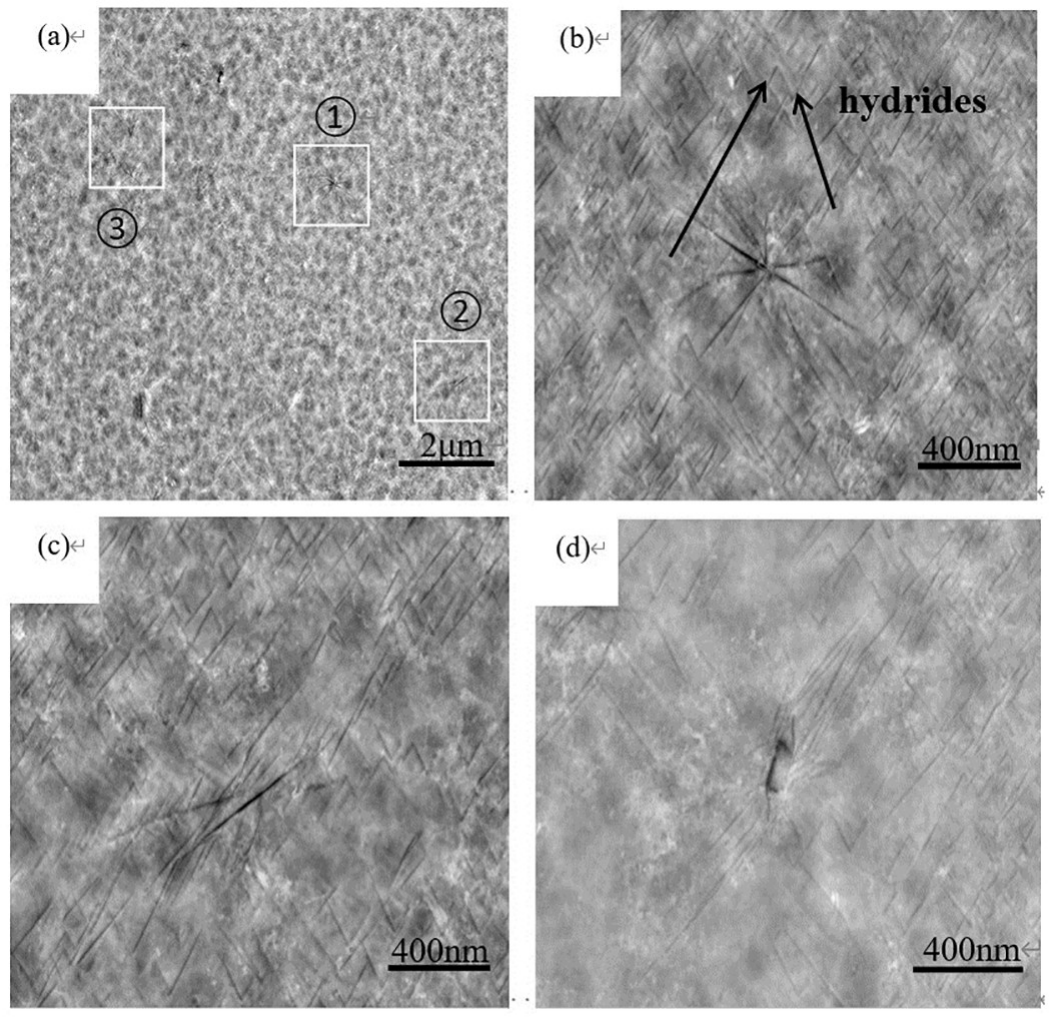
(a) EBSD-BSE microstructure of samples with hydrogen content of 0.18 wt%; (b) Enlarged view corresponding to ① in figure (a); (c) the enlarged image corresponding to ② in figure (a); (d) Enlarged view corresponding to ③ in figure (a).

Fig 4(a) presents the EBSD-BSE image of the TB8 sample with a hydrogen content of 0.18 wt%, while Fig 4(b)–4(d) provide magnified images of the corresponding regions in Fig 4(a). The image illustrates two distribution states of the hydrides: isolated and cluster forms, and the distribution of hydrides is uneven. These observations are consistent with the XRD results shown in Fig 3, which show a higher number of hydrides for TB8 alloys with a hydrogen content of 0.18 wt%.

**Fig 5:**
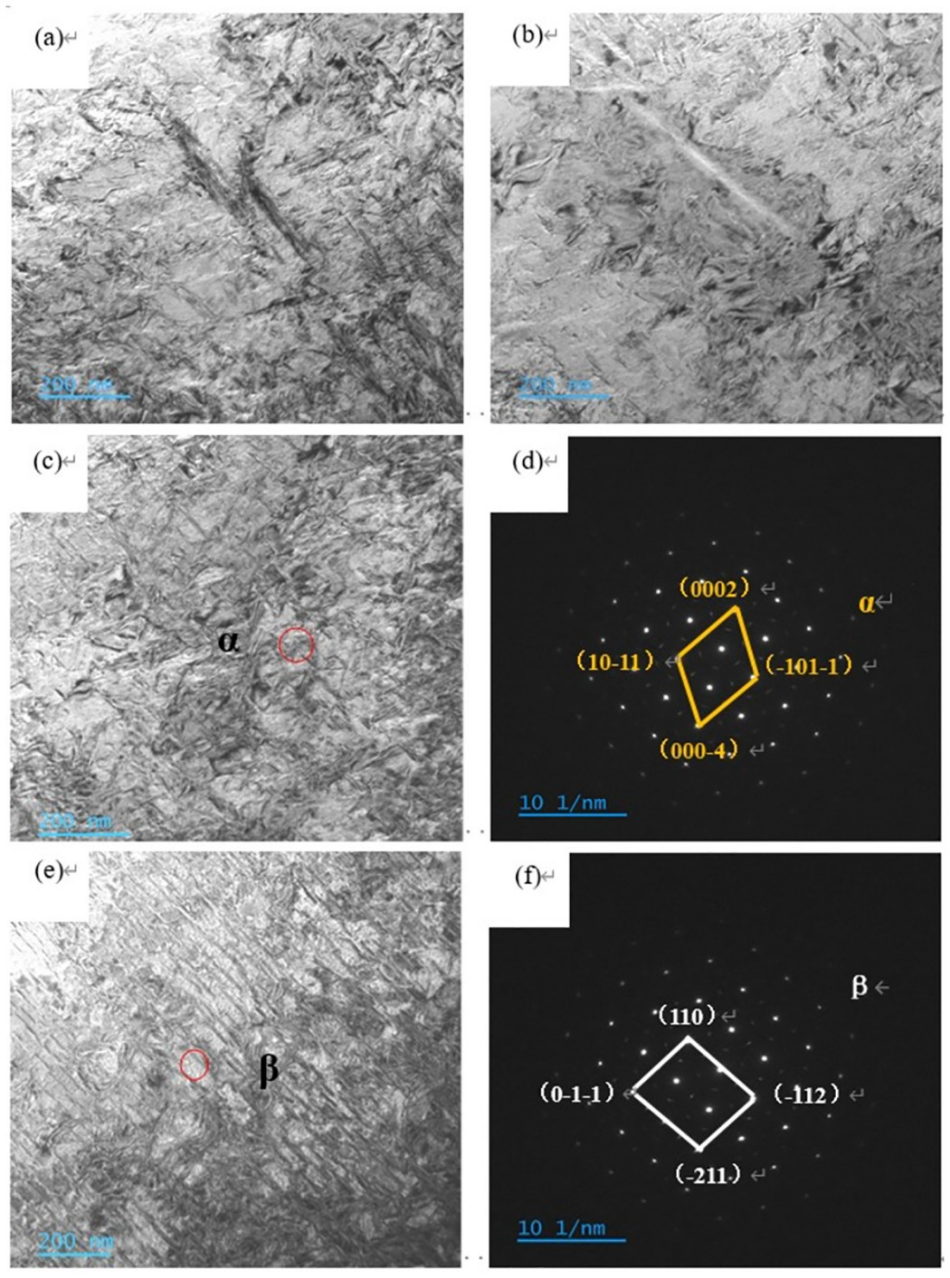
TEM morphology of (a), (b), (c), (e) TB8 alloy with hydrogen content of 0.18 wt%; (d) corresponding SAED pattern of α phase with [2110] zone axis; (f) corresponding SAED pattern of β phase with [1–11] zone axis.

Near-surface TEM samples of TB8 hydrogen charged samples with hydrogen content of 0.18 wt% are prepared by conventional grinding method and ion thinning. Fig 5(a)–5(c) and 5(e) show the TEM morphology. The SAED patterns of the α phase and β phase are shown in Fig 4(c) and 4(d). It can be observed that the alloy is mainly composed of β phase and a small amount of α phase. The α phase has a hexagonal close-packed (hcp) structure with [2110] as the zone axis, and the β phase has a body-centered cubic (bcc) structure with [1–11] as the zone axis.

### 3.2 Effects of hydrogen on the mechanical properties of the alloy

**Fig 6:**
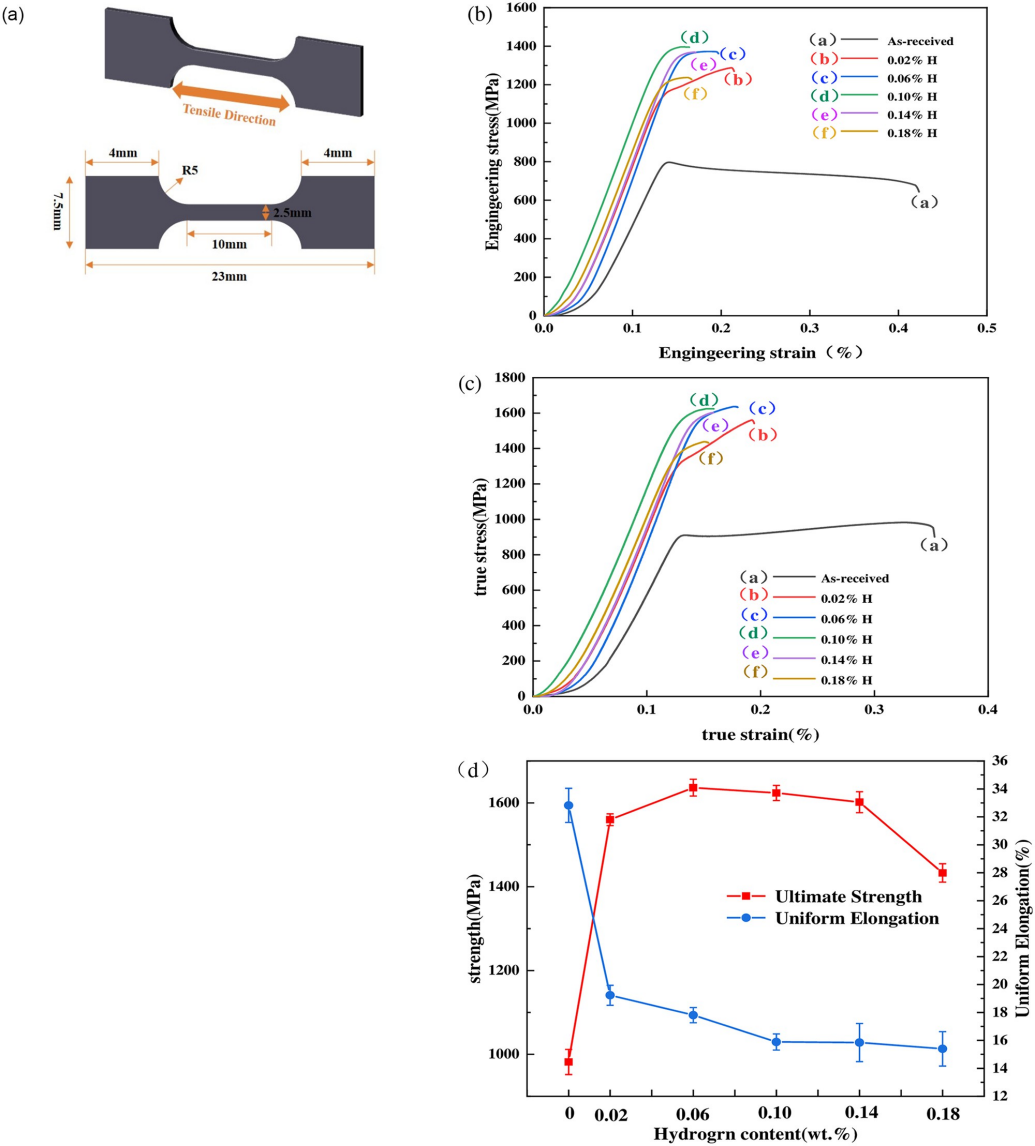
(a) The dimensions of the tensile sample. (b). Engineering stress-strain curves of the differently charged samples. (c) True stress-strain curves of the differently charged samples. (d) Uniform elongation and ultimately tensile strength (UTS) in different conditions.

The influence of hydrogen on the mechanical properties of the TB8 titanium alloy was examined through room temperature tension tests. Tensile tests were carried out using an LFM-20KN tensile testing machine at a strain rate of 1×10^-3^s^-1^. Fig 6 illustrates the sample dimensions, engineering stress-strain curves, and true stress-strain curves of samples with varying hydrogen content. Uniform elongation and ultimate tensile strength (UTS) of the uncharged and hydrogen-charged samples are list in Table 2.

**Table 2:** Mechanical properties of the samples after hydrogen charging for various contents.

Hydrogen contents (wt%)	UTS (MPa)	Uniform elongation(%)
0	981.89	32.82
0.02	1559.92	19.22
0.06	1636.23	17.80
0.10	1623.70	15.88
0.14	1601.73	15.84
0.18	1432.82	15.39

Fig 6(d) clearly demonstrates the effect of different hydrogen contents on the uniform elongation and ultimate tensile strength (UTS) of the TB8 titanium alloy. The uncharged TB8 sample exhibited obvious necking, indicating good ductility [32]. The UTS of the uncharged sample is approximately 982 MPa, with a uniform elongation of about 33%. When the hydrogen content is 0.02 wt% and 0.06 wt%, the UTS of the specimens increased to 1560 MPa and 1636 MPa, respectively. The XRD analysis suggests that at these hydrogen contents, less hydrogen and fewer hydrides are present. The hydrogen introduced into the alloy contributed to solid solution strengthening, thus increasing the alloy’s strength.

Nevertheless, with an increase in hydrogen content, UTS exhibited a gradual decline. This can be attributed to an increase in the presence of hydrides, especially when the hydrogen content reaches 0.18 wt%, the emergence of γ-TiH and ε-TiH_2_ hydrides results in the reduction of UTS. The precipitation of hydrides induced a ductile-to-brittle transition during tensile deformation, leading to premature fracture of the sample [33]. At this point, the UTS of the sample reached its lowest value at approximately 1432 MPa. The decrease in uniform elongation is also evident. For example, when the uncharged hydrogen content is 0.02 wt%, the uniform elongation decreases from 33% to 19%, and this trend persists as the hydrogen content increases. The decrease in elongation is associated with the transition from ductile to brittle fracture, as the number of hydrides increased with higher hydrogen content.

It can be seen from the results that as the hydrogen content increases, the UTS first increases and then decreases, but the UTS of the sample containing 0 wt% is also smaller than that of the sample containing 0.18 wt% hydrogen. The reasons for such an event from a physical point of view are presented as follows [34, 35]. Initially, when hydrogen is introduced into the titanium alloy, it forms an interstitial solid solution. This can lead to solid solution strengthening, where the presence of hydrogen atoms impedes the motion of dislocations within the crystal lattice. This, in turn, can increase the alloy’s UTS. AS the hydrogen content continues to increase, hydrogen can accumulate at grain boundaries and other defects in the material. When hydrogen is concentrated at these sites, it can embrittle the material, making it more prone to fracture. This is known as hydrogen embrittlement and can lead to a reduction in UTS.

### 3.3 Tensile fracture morphology analyses

**Fig 7:**
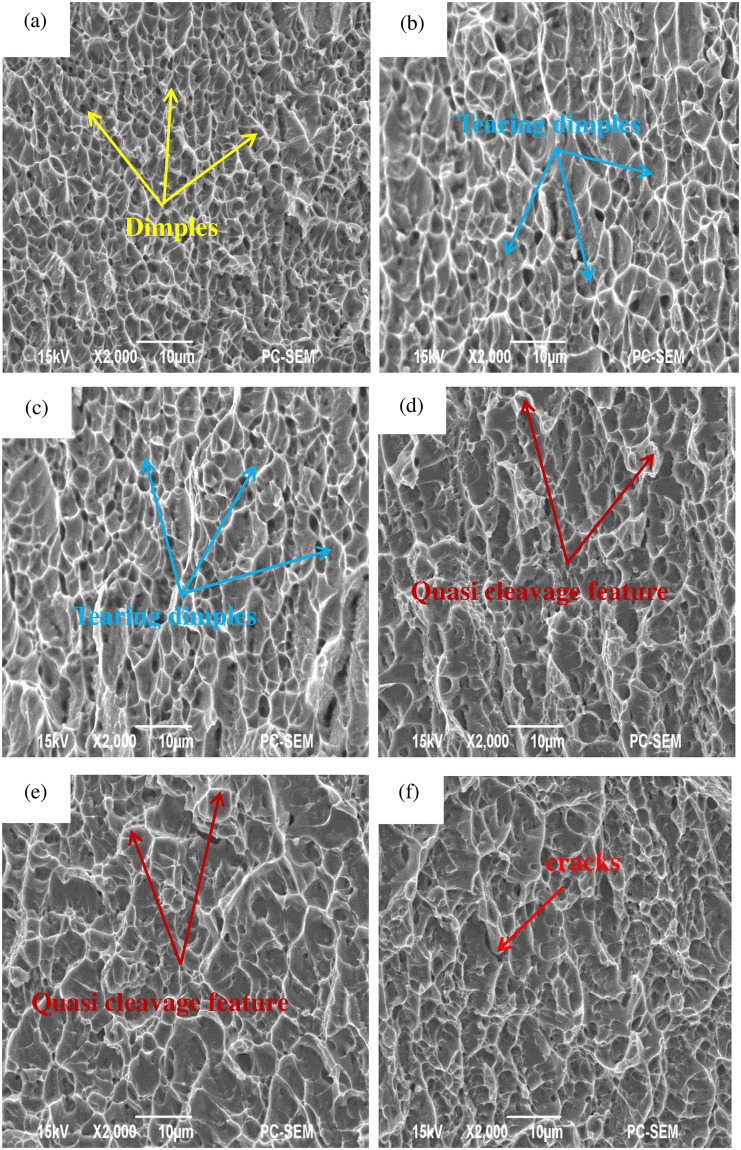
Fracture morphology in different conditions: (a) as-received. (b) 0.02 wt%. (c) 0.06 wt%. (d) 0.10 wt%. (e) 0.14 wt%. (f) 0.18 wt%.

To further analyze the fracture characteristics and explain the hydrogen-induced fracture mechanism, the fracture surfaces of the tensile samples were observed using SEM. Fig 7 illustrates the fracture morphology corresponding to the different tensile samples shown in Fig 6. Fig 7(a) reveals that the fracture surface of the uncharged sample is characterized by a significant number of ductile dimples, indicating ductile fracture and a fracture mode of ductile microvoid coalescence(MVC) [36, 37]. This observation aligns with the performance results of the uncharged sample in Fig 6(c), suggesting good plasticity of the alloy at this stage.

In Fig 7(b)–7(d), as hydrogen content increases, the fracture morphology exhibits numerous tearing tough nests and ridges, indicating a transition from ductile fracture to brittle fracture after hydrogen charging [29]. This change aligns with the significant decrease in uniform elongation observed in the alloy after hydrogen charging. At a hydrogen content of 0.14 wt%, quasi-cleavage features are observed, indicating a brittle fracture mode. At a hydrogen content of 0.18 wt%, Fig 7(f) demonstrates more pronounced quasi-cleavage features and the presence of cracks, further confirming a brittle fracture mode. Based on the XRD analysis and the results presented in Fig 3, it can be inferred that the increase in both the quantity and types of hydrides accelerates the fracture process, ultimately resulting in a decrease in the tensile strength and uniform elongation of the alloy.

### 3.4 Effects of hydrogen on the Vickers hardness of the alloy

**Fig 8:**
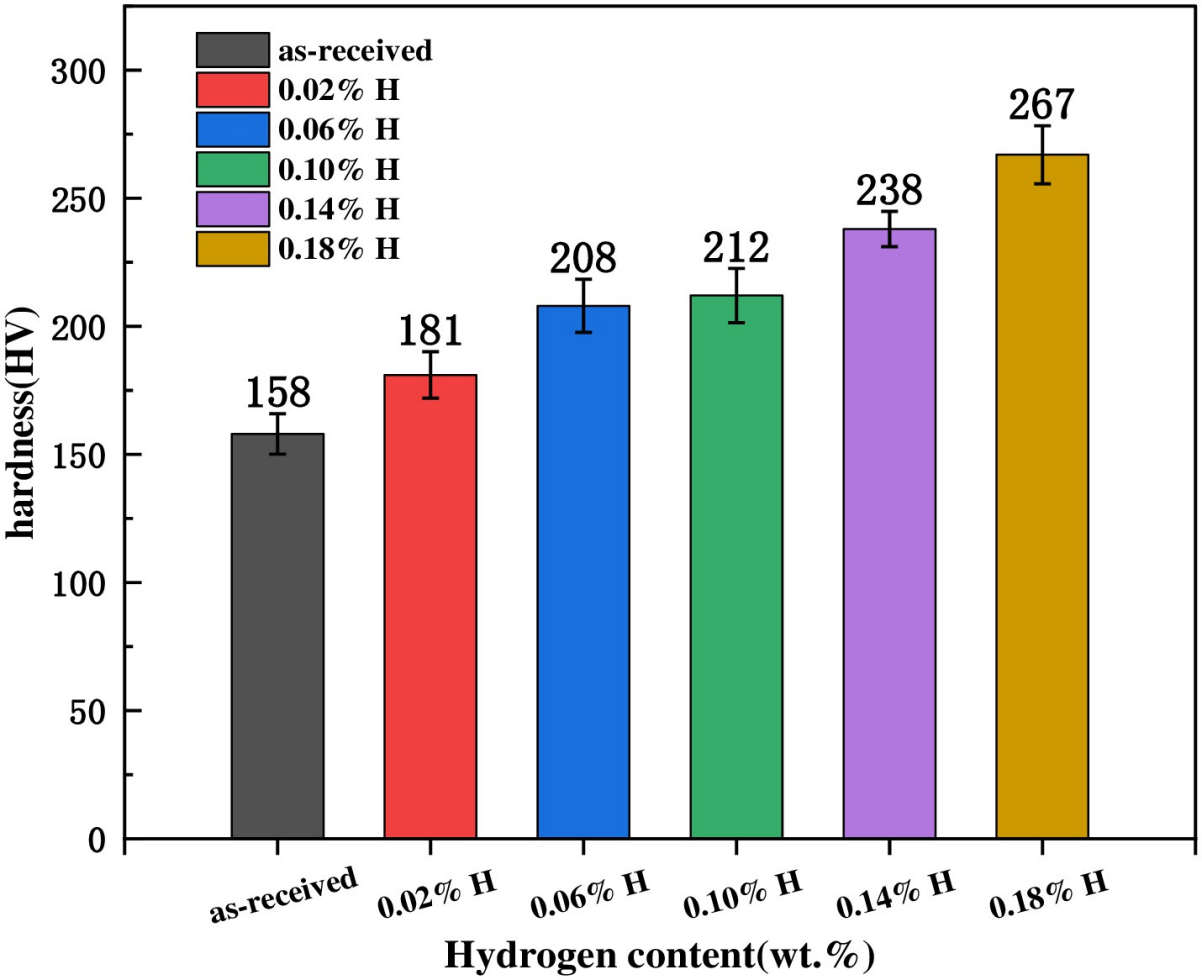
Micro-hardness of TB8 titanium alloy with various hydrogen charging conditions.

To investigate the influence of varying hydrogen contents on the Vickers hardness of TB8 titanium alloy, we conducted microhardness tests on hydrogen charged specimens using a microhardness tester. Fig 8 illustrates the relationship between alloy microhardness and hydrogen content. Notably, we observed a consistent increase in microhardness as the hydrogen content increased. Specifically, the Vickers hardness of the uncharged sample is 158HV, and when the hydrogen content is 0.18wt%, the hardness rises to 267HV. This trend reveals a positive correlation between alloy Vickers hardness and hydrogen content.

This observed phenomenon can be attributed to two primary factors. Firstly, the addition of hydrogen leads to its incorporation into the interstitial positions within the alloy matrix, resulting in the formation of an interstitial solid solution and a subsequent solid solution strengthening effect. As the hydrogen content increases, more hydrogen dissolves into the matrix, further augmenting the solid solution strengthening effect and consequently increasing the hardness of alloy [24, 38]. Secondly, as shown in the XRD analysis in Fig 3, upon initial hydrogen charging, the α phase of the matrix has a lower solubility for hydrogen due to its higher prevalence. This leads to hydride precipitation, causing lattice distortion and the generation of dislocations. These hydrides impede the movement of dislocations, resulting in increased hardness [39, 40].

Examining the metallographic structure in Fig 2, it becomes evident that hydrogen accumulates predominantly near grain boundaries in our study. Hydrogen exerts a pinning effect on dislocation motion, making dislocation sliding at grain boundaries less facile. Consequently, this leads to surface hardening of the titanium alloy [41, 42], contributing to the observed increase in hardness.

## 4. Conclusions

This study investigated the influence of varying hydrogen content on the microstructure, mechanical properties, and fracture behavior of metastable β titanium alloy TB8 after hydrogen charging. Tensile tests were conducted on both uncharged samples and samples with different hydrogen contents.

- The ultimate tensile strength of the alloy initially increased from 982 MPa to 1636 MPa and subsequently decreased to 1432 MPa, which can be attribute to the solid solution strengthening effect induced by the addition of hydrogen.
- Uniform elongation decreased from 33% to 19%. With the increase in hydrogen content, the depth and size of dimples on the fracture surface exhibited a decreasing trend, indicating a gradual deterioration of plasticity.
- The transformation from dimple fracture to quasi cleavage fracture was observed, suggesting that elevated hydrogen concentration contributed to increasing to hydrogen embrittlement sensitivity, thus leading to further deterioration of toughness.

These findings offer a valuable understanding of the microstructure evolution and mechanical properties changes associated with varying hydrogen contents in TB8 titanium alloy.
